# Linked open drug data for pharmaceutical research and development

**DOI:** 10.1186/1758-2946-3-19

**Published:** 2011-05-16

**Authors:** Matthias Samwald, Anja Jentzsch, Christopher Bouton, Claus Stie Kallesøe, Egon Willighagen, Janos Hajagos, M Scott Marshall, Eric Prud'hommeaux, Oktie Hassenzadeh, Elgar Pichler, Susie Stephens

**Affiliations:** 1 Section for Medical Expert and Knowledge-Based Systems, Center for Medical Statistics, Informatics, and Intelligent Systems, Medical University of Vienna, Vienna, Austria; 2 Information Retrieval Facility (IRF), Vienna, Austria; 3 Digital Enterprise Research Institute (DERI), National University of Ireland Galway, IDA Business Park, Lower Dangan, Galway, Ireland; 4 Web-based Systems Group, Freie Universität Berlin, Berlin, Germany; 5 Entagen, LLC, Second Floor, 44 Merrimac Street, Newburyport, MA 01950, USA; 6 H. Lundbeck A/S, Copenhagen, Denmark; 7 Department of Pharmaceutical Biosciences, Uppsala University, Uppsala, Sweden; 8 Department of Medical Informatics, Stony Brook University School of Medicine, Stony Brook, New York, USA; 9 University of Amsterdam, Amsterdam, The Netherlands; 10 Leiden University Medical Center, Leiden, The Netherlands; 11 W3C, Cambridge, MA, USA; 12 Department of Computer Science, University of Toronto, Toronto, Ontario, Canada; 13 W3C HCLSIG. W3C, Cambridge, MA, USA; 14 Johnson & Johnson Pharmaceutical Research & Development, L.L.C., Radnor, USA

## Abstract

There is an abundance of information about drugs available on the Web. Data sources range from medicinal chemistry results, over the impact of drugs on gene expression, to the outcomes of drugs in clinical trials. These data are typically not connected together, which reduces the ease with which insights can be gained. Linking Open Drug Data (LODD) is a task force within the World Wide Web Consortium's (W3C) Health Care and Life Sciences Interest Group (HCLS IG). LODD has surveyed publicly available data about drugs, created Linked Data representations of the data sets, and identified interesting scientific and business questions that can be answered once the data sets are connected. The task force provides recommendations for the best practices of exposing data in a Linked Data representation. In this paper, we present past and ongoing work of LODD and discuss the growing importance of Linked Data as a foundation for pharmaceutical R&D data sharing.

## Findings

Pharmaceutical research has a wealth of available data sources to help elucidate the complex biological mechanisms that lead to the development of diseases. However, the heterogeneous nature of these data and their widespread distribution over journal articles, patents and numerous databases makes searching and pattern discovery a tedious and manual task. From the perspective of a pharmaceutical research scientist, the ideal data infrastructure should make it easy to link and search across open data sources in order to identify novel and meaningful correlations and mechanisms. In this paper, we present work from the Linked Open Drug Data (LODD) task force of the World Wide Web Consortium (W3C) Health Care and Life Science Interest Group (HCLS IG) that aims to address these issues by harnessing the power of new web technologies.

The LODD task force works with a set of technologies and conventions that are now commonly referred to as *Linked Data*. The primary goal of the Linked Data movement is to make the World Wide Web not only useful for sharing and interlinking documents, but also for sharing and interlinking *data *at very detailed levels. The movement is driven by the hypothesis that these technologies could revolutionize global data sharing, integration and analysis, just like the classic Web revolutionized information sharing and communication over the last two decades.

Linked Data is based on a set of principles and standard recommendations created by the W3C. Single data points are identified with Hypertext Transfer Protocol (HTTP, [1]) Uniform Resource Identifiers (URIs). Similar to how a Web page can be retrieved by resolving its HTTP URI (e.g., 'http://en.wikipedia.org/wiki/Presenilin'), data about a single entity in the Linked Data space can be retrieved by resolving its HTTP URI (e.g. 'http://dbpedia.org/resource/Presenilin'). However, instead of Web pages, the primary data model of Linked Data is the Resource Description Framework (*RD*F, [2]). In RDF, entities, their relations and properties are described with simple subject-predicate-object *triples*. Out of these simple triples, sophisticated networks of interlinked data can be built, potentially spanning over several different locations on the web. Since every entity in this network can be resolved through HTTP, it is possible to navigate and aggregate the globally distributed data, enabling the important features of transparency and scalability that made the Web successful.

There is a large array of other standard recommendations based on RDF. Networks of RDF data can be queried by an intuitive and powerful query language called *SPARQL *[3]. The Web Ontology Language (*OWL*, [4]) makes it possible to do complex logical reasoning and consistency checking of RDF/OWL resources. These reasoning capabilities can be used to harmonize heterogeneous data structures. Another related standard is *RDFa *[5], which makes it possible to embed RDF statements into human-readable Web pages, effectively bridging the domains of human-readable and machine-readable data. Chen at al. provide an extensive review of RDF/OWL - based projects relevant to drug discovery in a recent publication [6].

To date, participants of the LODD project have made twelve open-access datasets relevant to pharmaceutical research and development available as Linked Data (table 1). These are DrugBank [7], ClinicalTrials.gov [8,9], DailyMed [10], ChEMBL [11,12], Diseasome [13], TCMGeneDIT [14,15], SIDER [16], STITCH [17], the Medicare formulary and the three most recent additions, RxNorm [18], Unified Medical Language System (UMLS, [19]) and the WHO Global Health Observatory [20]. To be kept up to date, the original datasets are periodically retrieved and the Linked Data representations are refreshed. The URIs for representing entities in the linked datasets are stable and are chosen by the LODD participants.

**Table 1:** The current LODD datasets.Further information about content and accessibility (URIs, SPARQL endpoints) of these linked datasets can be found online at [27].

Name	Short Description	Size and coverage (rounded)	Sources	Provider (1. original dataset, 2. RDF version of dataset)
DrugBank	Chemical, pharmacological and pharmaceutical drug data; data about drug targets (e.g., sequences, structure, pathways)	767,000 triples; 4,800 drugs, 2,500 protein sequences	Aggregated from various biomedical and pharmaceutical databases	1. University of Alberta 2. Free University of Berlin

ClinicalTrials.gov/LinkedCT	Information about clinical trials	9.8 million triples, 80,000 trials	Data submitted by study sponsors or their representatives	1. US National Institute of Health 2. LinkedCT.org; University of Toronto

DailyMed	Information about approved prescription drugs, including FDA approved labels (package inserts)	164,000 triples; 4,000 drugs	Package inserts, data from the US food and drug administration (FDA)	1. US National Library of Medicine 2. Free University of Berlin

ChEMBL	Information on drugs, e.g., activity against drug targets such as proteins, chemical properties. Linked to primary literature	24 million triples; 8000 drug targets, 660,000 compounds	Aggregated from various biomedical and pharmaceutical databases	1. European Bioinformatics Institute 2. Uppsala University

Diseasome	Characteristics of disorders and disease genes linked by known disease-gene associations	91,000 triples; 2,600 genes	Generated from data in *Online Mendelian Inheritance in Man *(OMIM)	1. Consortium of several labs 2. Free University of Berlin

TCMGeneDIT/RDF-TCM	Gene-disease-drug associations mined from literature about Chinese medicine	117,000 triples	Mined from research articles	1. National Taiwan University 2. Oxford University

RxNorm	Prescription drugs, their ingredients, and national drug codes	7.7 million triples; 166,000 unique drugs and ingredients	FDA databases	1. US National Library of Medicine 2. Stony Brook School of Medicine

UMLS	Unified Medical Language System (UMLS) sources available without restrictions	55 million triples	Ontologies created by third parties	1. US National Library of Medicine 2. Stony Brook School of Medicine

SIDER	Reported adverse effects of marketed drugs	193,000 triples; 63,000 adverse effect reports	Mined package inserts	1. European Molecular Biology Laboratory, Heidelberg 2. Free University of Berlin

STITCH	Molecular interactions between chemicals and proteins	7.5 million chemicals, 500,000 proteins, 370 organisms	Aggregated from various biomedical and pharmaceutical databases	1. European Molecular Biology Laboratory, Heidelberg 2. Free University of Berlin

Medicare	The Medicare formulary	44,500 triples; 6800 drugs	Primary data	1. US Government 2. Free University of Berlin

WHO Global Health Observatory	Data and statistics for infectious diseases at country, regional, and global levels.	354,000 triples	Primary data collected by the World Health Organization	1. World Health Organization 2. Leipzig University

Statistics about size and coverage were last checked on March 24, 2011.

Not all of these datasets can currently be considered fully 'open' as outlined by the Panton Principles [21]. For example, some of the source have non-commercial clauses in the license agreement. The LODD project is actively exploring the exact conditions for modification and redistribution defined by the data providers, and acknowledges the limitations with respect to openness some of these datasets currently have.

**Figure 1:**
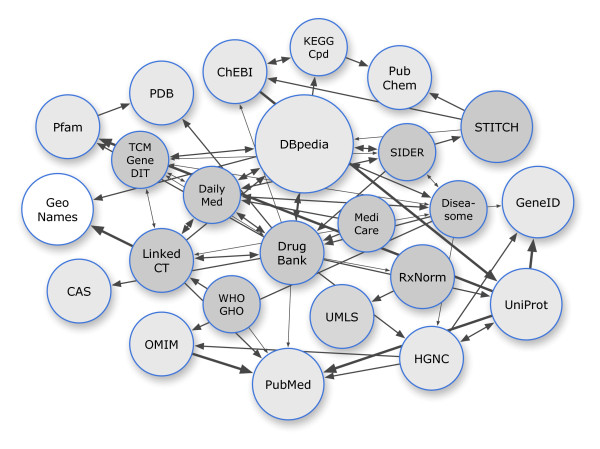
**A graph of some of the LODD datasets (dark grey), related biomedical datasets (light grey), related general-purpose datasets (white) and their interconnections**. Line weights correspond to the number of links. The direction of an arrow indicates the dataset that contains the links, e.g., an arrow from A to B means that dataset A contains RDF triples that use identifiers from B. Bidirectional arrows usually indicate that the links are mirrored in both datasets.

The LODD datasets are linked with each other, as well as with datasets provided by other Linked Data projects, such as Bio2RDF [22] and Chem2Bio2RDF [23], as well as primary data providers that offer their resources in RDF, such as UniProt [24,25] and the Allen Brain Atlas [26]. The links between datasets are depicted in Figure 1. Overall, there are several dozens of biomedically relevant linked datasets available to date.

**Figure 2:**
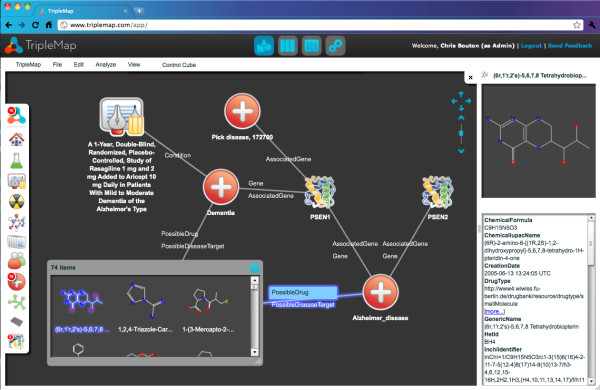
**TripleMap **www.triplemap.com** is a web-based application that provides a rich, dynamic, visual interface to integrated RDF datasets such as the LODD. On the left hand side of the application a researcher uses an icon-based menu representing biomedical entities such as compounds, diseases and assays to search for entities and view their associations**. Entities can be dragged and dropped from the icon menu into the application's zoomable workspace. In the middle of the application the user navigates maps of entities and their associations in the zoomable workspace much like users of Google Maps are able to scan and zoom into and out of geographically based maps. On the right hand side of the application the user can view an integrated set of all of the available properties for a selected entity. As entities are added to the workspace the system automatically generates semantically tagged edges between associated entities.

While the number of linked biomedical datasets has grown significantly over the last years, there is still a marked lack of mature applications that enable end-users to explore and query these datasets. Linked data browsers such as Marbles [28] or Sig.ma [29,30] are currently too generic for most end-users (although they can be very helpful for developers). These shortcomings are addressed by TripleMap (Figure 2,[31]), a new web-based application that can be used for the navigation, visualization and analysis of the LODD resources and other RDF datasets. To illustrate the use of TripleMap and the LODD resources, the following simple scenario could be imagined: A researcher interested in Alzheimer's Disease decides to find out everything that they can about the disease by querying an integrated version of the Linking Open Drug Data (LODD) sets. They open TripleMap and start their search by typing "Alzheimer's" into the Diseases search box. As they type, TripleMap provides a dynamic auto-complete list of all of the disease related entities across all LODD data sets that match the search string. The researcher selects "Alzheimer's Disease" and drags and drops it into the TripleMap workspace. Now, the researcher can view a range of information known about the properties of the disease in the right-hand "properties panel" including links out to Pubmed, Online Mendelian Inheritance in Man (OMIM, [32]), Uniprot [24] and other sources. These sources provide the user with rapid access to overview information about the disease.

The researcher is now interested in discovering entities that are associated with Alzheimer's Disease. They select the Alzheimer's Disease icon in the workspace and the system automatically shows them a number of associated disease genes provided by Diseasome, compounds provided by DrugBank and DailyMed, and clinical trials provided by LinkedCT. The researcher starts to explore relationships between entities by selecting two genes, presenilin (PSEN1) and amyloid precursor protein (APP), and dragging them into the workspace. In addition to finding genes related to Alzheimer's Disease, the user is interested in compounds known to be related to the disease. The user finds several compounds and pulls them into the workspace. The user is also interested in finding out what clinical trials are currently being run for Alzheimer's Disease and the system shows 200 such trials. With a simple click and drag action they pull all 200 trials into the workspace. As entities are added to the workspace, if there are known associations between them, those associations are also shown to the user as semantically tagged edges. This ability to show a researcher unexpected associations between entities that are related to their field of interest is at the heart of the value of an application like TripleMap and the extensive, rich, interconnected data available in the LODD data sets.

Linked Data as an emerging technology is still not free from shortcomings. A major problem is the heterogeneity in how data is modeled. Even when the entities between datasets are mapped to each other, it can still be difficult to intuitively write queries that span datasets because of this heterogeneity. This problem is being addressed by another task force of the W3C HCLSIG, which aims to bridge the data in the growing number of LODD datasets with a well-engineered top-level ontology, the translational medicine ontology (TMO, [33]). Another problem is how to efficiently query RDF in distributed SPARQL databases without requiring the aggregation of RDF data at a central location. Again, this is addressed by ongoing work on query federation by members of the W3C HCLS IG [34]. Finally, there has been a lack of applications with good user interfaces to make Linked Data resources accessible to end-users outside the biomedical informatics community. This is addressed by several ongoing endeavors such as the European Khresmoi project [35].

A challenge to creating linked data that is specific to the domain of chemistry is the provision of chemical identifiers. It is for this reason that W3C HCLS IG supports efforts to standardize unique identifiers for chemical compounds such as the IUPAC International Chemical Identifier (InChI, [36]).

The pharmaceutical industry is starting to embrace Linked Data with examples of projects being presented by Eli Lilly, Johnson & Johnson and UCB Pharma. While the adoption of Linked Data is still not yet very widespread in individual companies, it is on the agenda of several large-scale cross-pharma projects. An European project, the Open Pharmacological Space (OPS) Open PHACTS (Pharmacological Concept Triple Store) project under the European Innovative Medicines Initiative (IMI, [37]) wants to create an open source, open standards and open access infrastructure to enable integration of chemical and biological data to support drug discovery. The project intends to reach this goal by using Linked Data and managing the data in an RDF triple store. Collaboration across several IMI projects should also encourage the coordinated use of Linked Data to enhance data sharing. On the pre-competitive data sharing side of pharmaceutical informatics, the members of the Pistoia Alliance [38] are developing the Semantically Enriched Scientific Literature (SESL) project. The goal of SESL is to test the feasibility of executing federated querying across full text literature and bioinformatics databases by performing SPARQL queries on a triple store of assertions from the chosen data sources. The PRISM Forum [39] has also issued a letter recommending the adoption of Linked Data that has been supported by its membership of 15 of the top 20 pharmaceutical companies. The European OpenTox project [40,41] uses RDF as a standard for the exchange of predictive toxicology related data. The OpenTox framework defines algorithms, models, data sets, and chemical compounds, in a distributed data storage and computing facility.

Proprietary systems for providing integrated pharmaceutical data exist. The Accelrys/Symyx products [42] are popular examples, and can be both accessed online or installed locally. Accessing the data provided by these products often requires proprietary tools and internal installations also require ongoing work to be kept up-to-date. Furthermore, many of these products are based on individual databases that are not linked. Since the amount of data and the number of potential data sources is growing, it will become harder for single software vendors to create all-encompassing solutions. The nascent Linked Data infrastructure could help to make the creation of integrated solution more sustainable, easier to maintain and vendor-neutral.

Over the next years, the LODD group will continue to work jointly with both academic and industry partners. It will aim to become an umbrella for other Linked Data providers and consumers in the pharmaceutical domain, assisting with documentation, interlinking, quality management, and compliance with standard formats and vocabularies. Another strand of work will focus on how to integrate public Linked Data with non-public, in-house datasets of biomedical research institutions and pharmaceutical companies.

The LODD task force is open to new participants and interested individuals or groups are invited to get in contact with the authors of this paper.

## Competing interests

CB declares association with Entagen, LLC, a for-profit company that is building commercial software for semantic technologies such as TripleMap. All other authors declare no competing interests.

## Authors' contributions

MS wrote major parts of the manuscript and organized the paper writing process. AJ converted several of the LODD datasets. CB developed the TripleMap software. SS organized the Linked Open Drug Data task force. All authors participated in discussions and developments of the Linked Open Drug Data task force of the W3C Health Care and Life Science Interest Group. All authors read and approved the final manuscript.
